# Deep Hypersaline Anoxic Basins as Untapped Reservoir of Polyextremophilic Prokaryotes of Biotechnological Interest

**DOI:** 10.3390/md18020091

**Published:** 2020-01-30

**Authors:** Stefano Varrella, Michael Tangherlini, Cinzia Corinaldesi

**Affiliations:** 1 Department of Materials, Environmental Sciences and Urban Planning, Polytechnic University of Marche, 60131 Ancona, Italy; s.varrella@univpm.it; 2 Stazione Zoologica Anton Dohrn, Villa Comunale, 80121 Napoli, Italy; michael.tangherlini@szn.it

**Keywords:** marine prokaryotes, microbial diversity, polyextremophiles, deep hypersaline anoxic basins, blue biotechnologies, extremozymes, polyextremophiles, limits of life

## Abstract

Deep-sea hypersaline anoxic basins (DHABs) are considered to be among the most extreme ecosystems on our planet, allowing only the life of polyextremophilic organisms. DHABs’ prokaryotes exhibit extraordinary metabolic capabilities, representing a hot topic for microbiologists and biotechnologists. These are a source of enzymes and new secondary metabolites with valuable applications in different biotechnological fields. Here, we review the current knowledge on prokaryotic diversity in DHABs, highlighting the biotechnological applications of identified taxa and isolated species. The discovery of new species and molecules from these ecosystems is expanding our understanding of life limits and is expected to have a strong impact on biotechnological applications.

## 1. Introduction 

Deep-sea ecosystems (waters and seabeds of the ocean beneath 200 m depth) are the largest, most remote, and least explored biomes of the biosphere, comprising more than two-thirds of the oceanic volume [1,2,3]. They are characterized by absence of light, an average depth of approximately 4200 m, temperatures below 4 °C, and a hydrostatic pressure of about 40 MPa; taken together, these factors encompass some of the harshest environments on our planet, representing a challenge for the existence of life [2]. Over the last few decades, many deep-sea surveys have resulted in the discovery of highly diversified and peculiar habitats [2,4,5,6], including hydrothermal vents, cold seeps, mud volcanoes, and deep hypersaline anoxic basins, where life conditions are even more extreme [7]. Among these, deep hypersaline anoxic basins (DHABs) are defined as polyextreme ecosystems [8,9].

**Figure 1:**
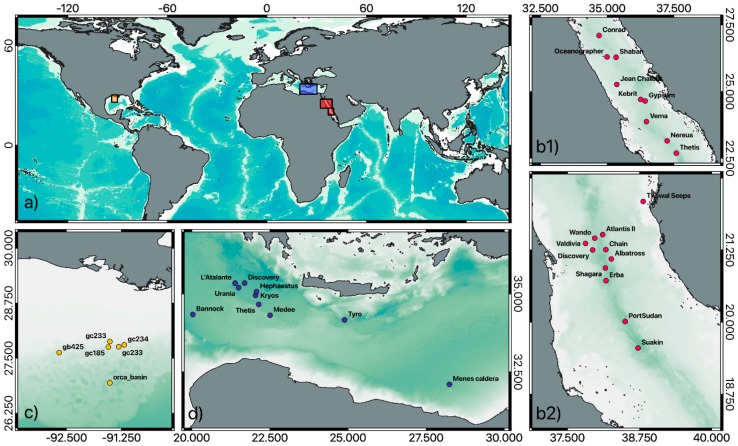
Global distribution of deep hypersaline anoxic basins (DHABs) (**a**). Locations and corresponding names of DHABs identified in the Red Sea (**b1**–**b2**), the Gulf of Mexico (**c**), and the Mediterranean Sea (**d**).

DHABs were discovered at the end of the last century on the seafloor in different deep-sea areas (at depths ranging from 630 m to 3580 m) around the globe (Figure 1), including the Mediterranean Sea [10,11], the Red Sea [12,13,14] and the Gulf of Mexico [15]. Intriguingly, the discovery of new DHABs is still ongoing, such as with the recent discovery of the new Thetis, Kyros, and Haephestus basins in the Mediterranean Sea [16,17,18]. To date, with the recent finding of these new DHABs, 35 basins have been discovered around the world. The Bannock, Tyro, Urania, L’Atalante, and Discovery basins are the deepest known DHABs, being far below the photic zone (3200–3500 m deep), and are located along the Mediterranean Ridge in the Eastern Mediterranean Sea, an accretionary complex subjected to continental collision [19]. Two of the most studied DHABs in the Red Sea are the Shaban and the Kebrit deeps. The Shaban Deep comprises four depressions at a depth of 1325 m, whereas the Kebrit Deep is a rounded basin of approximately 1 km in diameter found at a depth of 1549 m [20].

Different sampling strategies have been adopted to explore the general physical structure of DHABs. For instance, Mediterranean DHABs have been sampled through a rosette with Niskin bottles equipped with a conductivity, temperature, and depth (CTD) sensor and connected to a live camera to monitor the sampling operations [21,22,23]. Geochemical data of DHABs, such as those located in the Gulf of Mexico, have been collected through a brine-trapper, which was used to collect vertically water from different layers of the seawater–brine interface [24].

**Figure 2:**
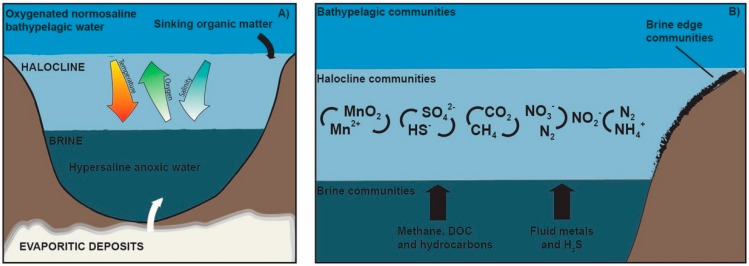
Simplified vertical section of a DHAB. The transition from the overlying seawater to the brine is commonly referred to as the halocline or brine–seawater interface, which is characterized by gradients of temperature, salinity, pH, and dissolved oxygen (**A**); the main biogeochemical processes taking place within the halocline are shown in (**B**). From left to right, the manganese cycle, the sulfate reduction and sulfide oxidation cycle, the methanogenesis and aerobic (anaerobic) methane oxidation cycle, and the anammox and denitrification cycle that occur in the halocline are shown [8,21,23,28,29,30]. DOC: Dissolved Organic Carbon.

Despite the different geological features found in DHABs, most of them are derived from the re-dissolution of evaporitic minerals, like halite (NaCl-mineral) and kieserite (MgSO_4_-mineral), after exposition to seawater due to tectonic activity [25,26]. This determines a salt-induced stratification of the water column (Figure 2), which drives the formation of a stable, dense, hypersaline brine lake with a variable thickness, ranging from one to tens of meters; this brine lake represents a polyextreme environment because its conditions hinder oxygen exchange, creating euxinic conditions, including high hydrostatic pressure, extremely low water activity and chaotropicity, and sharp oxy-, picno-, and chemoclines at the seawater–brine interface [21,23,27]. The salt concentration progressively increases over depth in the overlying halocline interface, reaching brines values up to 7–10 times higher than those existing in seawater [28].

Chemical and physical characteristics are specific to each DHAB and greatly vary depending on how the brine was formed along with the geographic localization (Table 1). The majority of the DHABs are thalassohaline (most of the dissolved ions are represented by those composing the overlaying seawater), whereas the Discovery, Kryos, and Hephaestus basins are athalassohaline and are characterized by high Mg^2+^ concentrations likely deriving from the dissolution of magnesium chloride salts (i.e., bischofite [18])

Overall, DHABs can be subdivided into four different systems: the seawater–brine interface, brines, the brine–sediment interface, and the sediments underlying the brines. Each of these features is characterized by specific conditions such as the steep halocline at the water–brine interface or the anoxic conditions of the sediments beneath the brines. In addition, the geochemical characteristics of each DHAB are mostly dependent on their geological evolution and origin. The high density of the brine prevents their mixing with the overlying oxygenated seawater, thus making the DHABs completely anoxic [16]. Their different hydrochemistry and physical separation for thousands of years has made these systems greatly interesting for scientists due to their potential similarity with extraterrestrial environments [18,34,37]. Despite their extreme conditions, many studies have provided evidence of a highly active prokaryotic community and of the presence of living metazoans, greatly extending our knowledge regarding the limits of organisms’ adaptions to life [20,27,38,39,40,41,42,43,44,45,46,47,48]. These organisms require specific adaptations for withstanding numerous physicochemical stresses [49].

The complex structure and conditions of the DHABs, such as the presence of the steep halo- and oxyclines, have been found to influence the distribution, structure, and richness of the microbial communities living in these environments [19]. Many studies have been focused on the halocline, which entraps nutrients, sinking organic materials, minerals, and microbial cells, and creates environmental gradients of great interest not only for identifying and isolating novel organisms but also for clarifying their metabolic strategies employed for adapting to extreme conditions [19]. The variable accumulation of metals and nutrients, especially in the halocline, supports the presence of different ecological niches exploited by highly diverse microorganisms with peculiar features [38]. However, to date, our knowledge of how these organisms are affected and contribute to the geochemical properties of the DHABs is still limited. 

The presence of life in these extreme environments has raised important questions about the molecular mechanisms that extremophiles have developed to overcome harsh conditions. Many studies have highlighted several peculiar adaptive strategies of halophilic microorganisms for maintaining stability and functionality of all their cellular components under such conditions [50,51]. Hence, microorganisms inhabiting extreme saline habitats not only have been considered useful subjects for ecological and evolutionary studies [50] but also hold an outstanding ability to produce bioactive molecules and enzymes, which can also be exploited for industrial and biotechnological purposes as well as for human wellness [52,53]. Considering the promising biotechnological potential of bacteria and archaea from DHABs due to their capability to live under extreme conditions, the present review provides an outline of the prokaryotic biodiversity in DHABs, highlighting their potential in producing enzymes and bioactive molecules for industrial, pharmaceutical, and environmental applications. 

## 2. Prokaryotic Assemblages of DHABs

The specific characteristics and geochemical conditions of each DHAB have driven the development of different and highly-stratified communities. Brines and the seawater–brine interfaces, indeed, represent the most widely-studied domains within DHABs from both a taxonomic and ecological/functional point of view [21,23]. The halocline is a microbial “hotspot”, harboring dense microbial populations that appear to be more metabolically active than those of the adjacent layers, with the presence of unique bacterial lineages having been found [21,22,44,54,55]. Several microbial lineages have been identified within DHAB brines. In particular, many members of the new Mediterranean Sea Brine Lake lineages (MSBL1–6) have been found extensively across hypersaline basins [56] from the Mediterranean to the Red Sea (despite their name), and include Archaea (e.g., MBSL1, which are sugar-fermenting organisms capable of autotrophic growth [23,56,57] and other major divisions of bacteria (MSBL2–6 [21,38]). Interestingly, the bacterial MSBL2 lineage has shown high similarity to the SB1 division found in the Shaban Deep brine pool, located in the Red Sea, which represents a novel halophilic lineage within bacteria, with no close cultivated relatives observed so far [28,38]. Similarly to the MBSL lineages, bacteria belonging to candidate division KB1 have been identified for the first time within the Kebrit Deep basin (Red Sea [20]) and subsequently have also been found in other DHABs of the Red Sea [58], as well as in other basins (e.g., the halocline of Mediterranean Sea brine pools and pools from the Gulf of Mexico [17,21,22,31,59]). Bacteria from this division can import and/or produce glycine betaine in response to osmotic stress [59]. The KB1 glycine betaine transport systems seem to aid not only in maintaining osmotic balance but also have a role in methane production [59]. Delta- and *Epsilonproteobacteria* are also widely distributed across DHABs. 16S rRNA gene libraries from the Bannock, Hephaestus, and L’Atalante basins (Mediterranean Sea) have provided evidence of the presence of sulfate-reducing *Deltaproteobacteria* (in particular belonging to the ANME-1 clade, responsible for the anaerobic oxidation of methane [16]) and sulfur-oxidizing Gamma- and *Epsilonproteobacteria* [21,22]. In the GC233 basin within the Gulf of Mexico, combining geochemical data and molecular analyses, different *Deltaproteobacteria* sulfate-reducers (related to *Desulfosarcinales*, *Desulfobacterium*, *Desulfobulbus*, and *Desulfocapsa*) and sulfide-oxidizing *Epsilonproteobacteria* have been found, leading to the hypothesizing of the presence of a sulfur-cycling microbial community [15,60]. 

Archaea associated with the ammonia-oxidizing *Thaumarchaeota* Marine Group I have also been found across several DHABs worldwide [18,21,22]. In particular, they have appeared to be the most representative prokaryotic members in different Red Sea DHABs, though with different contributions: in the Atlantis II and Discovery, 99% of archaeal operational taxonomic units (OTUs) were found to belong to the phylum *Thaumarchaeota*, whereas in the Erba basin the percentage was about 64% [30]. Members of this phylum are capable of fixing CO_2_ and oxidizing methane, contributing to dark primary production [61]. Overall, the dominant thaumarchaeal lineage is closer to the genus Nitrosopumilus [62]. The adaptation of this genotype to the hostile brine–sediment interface environment can be possible not only by increasing intra-cellular salt concentrations [63] but also for the presence of “acidic tuned” membrane proteins which show optimal activity and stability at high salinity [64]. Furthermore, genomic analyses have revealed the presence of specific pathways for taking up a mixture of osmolytes and other genes encoding for the biosynthesis of ectoine/hydroxyectoine, which are not present in mesopelagic clades [30]. However, different DHAB geochemistry may shape other thaumarchaeal lineages. Genomic analyses have revealed a newly isolated methanogenic archeon from the sulfide-rich halocline of Kebrit, which holds adaptive traits (e.g., osmoprotection and oxidative stress response) for counteracting the harsh local conditions [65].

Apparently, as high salinity is one of the main features of DHABs, halophilic organisms have been found across all the basins, and most of the isolated halophilic strains also display interesting metabolic features. In particular, 33 halotolerant bacterial strains have been isolated from the halocline of the Urania, Bannock, Discovery, and L’Atalante basins [66]. For instance, *Halanaeroarchaeum sulfurireducens* M27-SA2 is a sulfur-reducing and acetate-oxidizing haloarcheon isolated from the Medee basin [67]. Moreover, several novel strains have been isolated from Red Sea DHABs, such as *Halorhabdus tiamatea* (a non-pigmented, fermenting member of the *Halobacteriaceae* [68]) and *Haloplasma contractile* (a highly unusual contractile bacterium belonging to the Haloplasmatales order, which can grow under 0.2–3.1 M NaCl conditions [69]. Two other strains of a novel species, *Marinobacter salsuginis* SD-14BT and SD-14C, have also been isolated from the halocline of the Shaban Deep [70].

In general, prokaryotic diversity and activity appear to be less marked in sediments under brines than in deep-sea control sediments [55]. This is likely due to the cumulative physico-chemical stressors that greatly limit the survival of microorganisms which could be better adapted to the extreme DHAB chemocline [27,55,71]. Proteobacteria, Actinobacteria, Deferribacteres, and Euryarchaeota have been found in sediments underlying either Discovery Deep or Atlantis II [72]. In the sediments of L’Atalante, OTUs belonging to the *Pseudoalteromonas*, *Halomonas*, and *Pseudomonas* genera have been observed to be the most represented within the abundant Gammaproteobacteria class, suggesting a mixed assemblage of halophilic and halotolerant microorganisms [55]. In addition, metatranscriptomic analyses have revealed that, in the sediments underlying the Urania basin, most transcripts are affiliated with rRNAs of the genera *Pseudomonas*, *Rhodobacter*, and *Clostridium*, and with sequences associated with mitomycin antibiotics typically produced by *Streptomyces* [71]. Prokaryotes inhabiting DHAB sediments are killed by viruses, which may represent the main mechanism of top-down control of prokaryotic dynamics in these ecosystems [42]. Since viruses are found to be well-preserved in DHAB sediments, they can shape prokaryotic assemblages [41]. Based on this information, it is possible to hypothesize that prokaryotes of DHABs can produce specific molecules against viral infections.

## 3. Biotechnological Potential of Prokaryotes Inhabiting DHABs 

Generally, marine microorganisms represent an untapped source for the discovery and development of new biomolecules due to their rich biodiversity and genetic capacity to produce unique metabolites [73,74,75]. In this regard, it is well documented that many taxonomically novel marine species are promising sources of new bioactive compounds with noteworthy pharmaceutical activities, which can become sources of novel therapeutic agents [52,76]. In particular, marine extreme environments, like deep-sea and polar ecosystems or DHABs, have been revealed to be a rich source of secondary metabolites with novel structures and outstanding biological activities [28,52,77].

Due to the limited accessibility and remoteness of such extreme ecosystems and the need for sophisticated instruments for exploring and investigating them, they are still largely understudied and underexploited in comparison with terrestrial ecosystems.

**Figure 3:**
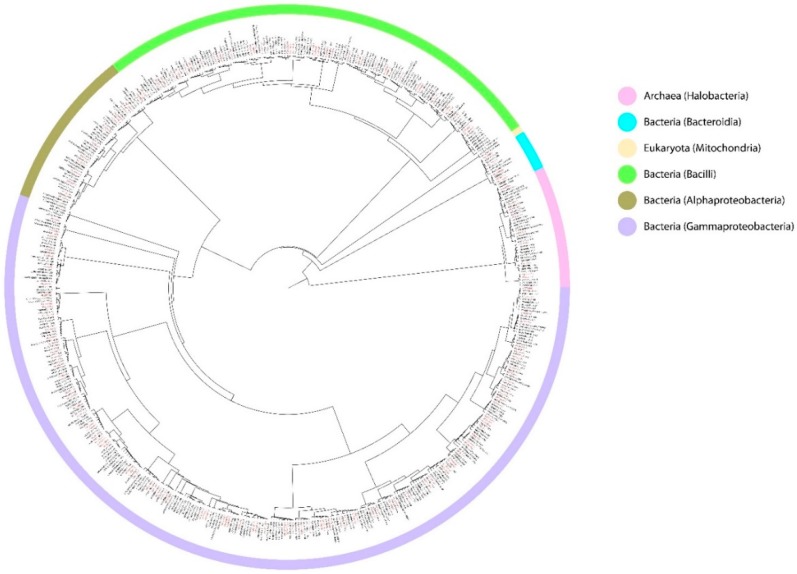
Phylogenetic tree of bacterial and archaeal strains isolated from DHABs. The tree was built using 16S rRNA gene sequences from [66,83,84,85] and phylogenetically close 16s rRNA sequences from the SILVA database v132.

Over the last few years, the advancement of technologies for deep-sea exploration [78] and “-omics” (e.g., environmental shotgun sequencing and metatranscriptomics) for the analysis of environmental strains of prokaryotes has revolutionized bioprospecting in extreme environments, thus increasing our knowledge of the genetic potential of microbial communities for the discovery of enzymes with a commercial value [79,80]. In addition, functional screening of extremophile metagenomes could represent a valuable approach to identify novel antibacterial and anticancer agents. In this regard, bioinformatic tools like the metabolite analysis shell (antiSMASH) have recently been used to detect from metagenomic samples collected from the Atlantis II, Discovery, and Kebrit DHABs promising specialized metabolism gene clusters (SMGCs) coding for products with reported antibacterial and anticancer effects, namely terpenes, peptides, polyketides, and phosphonates [81]. Two clones belonging to these libraries which exhibited antibacterial effects were screened by high-throughput sequencing (NGS) and bioinformatic analyses along with cytotoxicity assay (MTT) testing of the whole cell lysates against different cancer cell lines (MCF‑7, U2OS, and 1BR-hTERT) [82]. Although culture-independent approaches have radically changed microbial bioprospecting in extreme environments, the development of biotechnological applications must be accompanied by the corresponding study of pure cultures. In this regard, despite the great biodiversity-highlighted trough metagenomics in DHABs, so far less than 100 bacterial strains (Figure 3, Table S1) have been isolated and cultured for testing their extracts in few biotechnological applications [66,83,84,85]. Bioinformatic analyses on the phylogeny of the 16s rDNA sequences of those cultured strains (carried out by aligning them on the SILVA database v132 on the ACT server [86]) showed that most of these prokaryotes are affiliated with Gammaproteobacteria and Bacilli; in addition, several sequences within the same database were found to be phylogenetically related to the cultured strains (Table S2), further suggesting that more prokaryotic strains with adaptations to polyextreme ecosystems with biotechnological potential might be found within the same clades of already-cultured strains. Novel sampling and cultivation methods should be developed as alternatives to overcome culture limitations, especially in extreme environments [87].

Since the beginning of the new millennium, a number of studies have indicated the beneficial roles of extremophilic marine prokaryotes, which are a relevant but still underexplored source of bioactive molecules of commercial significance [74,88,89]. Extremophiles undoubtedly show unique capabilities and adaptations which allow them to thrive in systems characterized by harsh environmental conditions [90]. In fact, polyextremophilic microorganisms utilize alternative metabolic pathways and adaptive mechanisms which have important applications in industrial and environmental fields [50]. Since these microorganisms live in a biologically competitive environment for space and nutrients, they have developed mechanisms of defence against competitors and predators for their own survival, synthesising secondary metabolites of great value in pharmaceutical and biotechnological applications [91,92]. The advances in genome sequencing of extremophilic microorganisms have allowed us to provide a comprehensive understanding of their applications [93,94,95]. Moreover, microbes with large genomes, usually inhabiting complex harsh environments, can produce a vast array of secondary metabolites [96,97].

### 3.1. DHABs as a Hidden Treasure for Biodiscovery of Pharmaceuticals

Over the last 50 years, the development of new multidrug-resistant pathogens, along with the consequent increase in infectious diseases, has become an important issue for human wellness [98]. Furthermore, anticancer chemotherapeutic resistance is recently becoming a biomedical challenge, arising either intrinsically or extrinsically, after therapy [99]. Thus, the need for the discovery and development of novel antimicrobial and chemotherapeutic drugs with new modes of action is nowadays becoming of fundamental importance [100,101]. Since most of the antibiotics currently available on the market have been extracted from terrestrial organisms or derived semisynthetically from fermentation products, the isolation of microorganisms from marine habitats represent an interesting possibility which can lead to the discovery of novel structures with antibiotic activity [102,103]. 

As such, the prokaryotic genera identified in DHABs isolated from different marine environmental sources, including extreme environments, represent an authentic treasure of many bioactive compounds useful for biomedical applications (Table 2).

For instance, in Mediterranean DHABs transcripts related to *Streptomyces* have been identified, thus representing an important source of bioactive natural products with clinical or pharmaceutical applications [71,155]. Additionally, *Pseudoalteromonas flavipulchra* recently isolated from the Nereus halocline shows great antimicrobial activity which is associated with the different metabolites and/or enzymes that this species can produce [84,156,157].

An attractive example of the potential of extremophiles in the biomedical field has been provided by *Halobacteroides lacunaris* TB21, which was isolated from Thetis basin [118]. This polyextremophile organism produce a lipopolysaccharide (LPS) analog which can bind to the TLR4/MD-2 complex in HEK 293 hTLR4 cells, exerting an immunostimulant activity [118]. Additionally, *Pseudoalteromonas carrageenovora* isolated from Erba basin sediments is able to produce an LPS whose function is still undescribed [84,122]. This halophilic bacterium can also produce low molecular weight products from carrageenans, which have been reported to hold protective effects against the human immunodeficiency virus, the yellow fever virus, the herpes simplex viruses, the vaccine virus, and the pig fever virus [158]. Another interesting species isolated from the Nereus brine-pool–sea-water interface is *Bacillus halodurans,* which was engineered for the production of the anti-viral therapeutic peptide Enfuvirtide, marketed by Roche under the trademark Fuzeon^®^, which has given rise to possibilities for pharmaceutical applications [84,110]. In addition, *Zunongwangia profunda,* inhabiting the same interface, produces exopolysaccharides (EPS) with antioxidant properties [154]. 

Innovative research was carried out for screening the bioactivity of molecules extracted from the Atlantis II, Discovery, Kebrit, Nereus, and Erba DHABs [83,85]. Extracts from 36 isolates were tested on three different human cancer cell lines: HeLa, MCF-7, and DU145 [83,85]. In particular, many extracts from *Halomonas* strains have been found to induce apoptotic and cytotoxic effects. For example, *Halomonas meridiana* collected from Nereus halocline has been observed to prompt apoptosis of MCF-7 cells [85]. Recently, it was shown that the extract of *Pseudoalteromonas mariniglutinosa* collected from Erba and Nereus haloclines also inhibited the growth of MCF-7 cells [84]. *Halomonas* species can produce EPS which have been shown to have pro-apoptotic activity towards human T-leukemia cells and breast cancer MCF-7 cells [159,160]. Other bioactive extracts derived from *Chromohalobacter salexigens*, *Chromohalobacter israelensis*, *Halomonas meridiana*, and *Idiomarina loihiensis* have been found to be able to induce more than 70% mortality in HeLa cancer cells through different caspase-mediated apoptotic pathways [83]. 

Intriguingly, three extracts belonging to the genus *Salinivibrio* have been found to specifically blocked the growth of fibrosarcoma cells (HT-1080), opening up interesting perspectives for the discovery of new bioactive compounds produced by this genus [84]. The extracts of *Halomonas hamiltonii* and *Alcanivorax dieselolei* have been observed to inhibit the proliferation of BT20 cells, whereas the *Alteromonas macleodii* extracts collected from Nereus and Erba halocline were found to inhibit the cell growth of HCT [84]. *Alteromonas species* are also well known for producing dithiolopyrrolone, a potent antibiotic approved by the Food and Drug Administration and commercialized as Bactroban^®^ (GlaxoSmithKline) [104]. These studies emphasize the wide diversity of brine pool microorganisms capable of producing bioactive molecules, highlighting the incredible potential of DHABs as a source of novel molecules exploitable in the pharmacological industry.

### 3.2. DHABs as a Reservoir of Polyextreme Enzymes

Today’s society is moving toward “white” (i.e., industrial) biotechnology, which is growing for its efficiency from environmental and commercial points of view [161]. For example, natural enzyme catalysis has been utilized for application in a broader range of industrial settings, representing a valuable alternative to its chemical catalysts [162,163,164]. It is expected that 40% of the industrial application of chemical reactions that require organic solvents harmful to the environment will be replaced by enzymatic catalysis by 2030 [165]. The continuous demand for natural new enzymes that are biocompatible and non-toxic and which have high activity over a wide range of conditions, including temperature, salinity, pH, and metal concentrations, has been scaled up within pharmaceutical, food, and beverage industrial processes [166]. Hence, extremophilic microorganisms represent important sources of stable and valuable enzymes which are used as biocatalysts in industrial and biotechnological processes [53]. Enzymes from these organisms, which are called “extremozymes” due to their special features, can catalyze chemical reactions under conditions which inhibit or denature the non-extreme forms [167], including high salinity, acidic or basic pH, and high temperatures [168]. Thus, through the use of genetic engineering and/or by bioprospecting of extreme environments it is possible to discover and develop new extremozymes that can be suitable for many industrial processes [164]. Extremophilic bacteria and archaea produce enzymes which can be employed in industrial reactions using either directly living organisms or purified molecules, expanding the ranges of optimal enzyme performance and thus enabling biocatalysis under the enzymatically unfavourable conditions found in industrial processes [169]. Hence, the peculiar characteristics of extremophiles belonging to prokaryotic domains living in DHABs can represent a new source for exploitable enzymes for their capacity to operate under extreme conditions [170,171]. In fact, many of these molecules (e.g., aldehyde dehydrogenase, proteases, cellulases, esterases, ferredoxin oxidoreductase, agarase, amylases, κ-Carragenases, ketoreductases, and cyclodextrin glycosyltransferase) have been commercialized and have applications in different biotechnological areas with considerable benefits for many kinds of industries (Table 3). In particular, they are currently being employed in “red” biotechnology (i.e., biotechnology applied to pharmaceutical and medical fields). Other enzymes such as cellulase, chitinase, esterase, mercuric reductase, and β-glucosidases are exploited in “grey” (i.e., environmental) biotechnology while lipase is used in “blue” biotechnology, being applied to aquatic organisms and β-glucosidases and xylanase in biofuel production.

For instance, the production of novel thermoactive and alkali-tolerant α-amylases has been documented for many prokaryotic species such as *Pontibacillus chungwhensis*, *Halomonas meridiana*, and *Zunongwangia profunda* isolated taxa from DHABs. This group of enzymes has a very wide spectrum of industrial application, including in the sugar production, animal nutrition, baking, brewing, and distilling industries, in the production of digestive aids, in the pharmaceutical industries, and in the production of biofuel [239]. Amylases are consistently the most important among the enzymes of industrial interest and are forecasted to reach US$ 6.2 billion by 2020 [240]. For this reason, there is noteworthy attention paid to extremophilic α-amylases that have activity and stability characteristics suitable for the harsh conditions, including extreme salinity (2–4 M NaCl) and elevated temperature (80°C), demanded by industrial activities [241].

Interestingly, nitrilases have also been identified in DHABs, and are employed as commercial biocatalysts for the synthesis of plastics, paints, and fibers in the chemical industries and are also employed in the pharmaceutical industries for the manufacturing of (S)-ibuprofen, a widely used non-steroidal anti-inflammatory drug [242]. Moreover, nitrilases can detoxify cyanide present within wastes and degrade herbicides, representing an enzyme of extreme importance in bioremediation [243]. Biotransformation using native organisms as catalysts tends to be insufficient because the amount of nitrilases present as total cellular proteins is very low, and the reaction rate is slow and unstable [244]. Thus, the nitrilase recently identified by metagenomes mining in the Atlantis II DHAB could represent a valuable alternative not only for its thermal stability and tolerance to heavy metals compared to closely related nitrilases but also for the great number of microorganisms which could possess and produce these enzymes [211]. Another example of utilising sequence-based and activity-based metagenomics in mining for potential industrial biocatalysts is the esterase EstATII collected from the Atlantis II basin in the Red Sea, which displays a combination of extremophilic properties [197]. This enzyme is thermophilic (optimum temperature 65 °C) and halotolerant (for up to 4.5M NaCl) and maintains significant activity in the presence of a wide variety of toxic heavy metals, making it a potentially useful biocatalyst [197]. In agreement with this study, O.16 esterase was identified in the Urania basin which shoswed remarkable polyextremophilic properties (i.e., 180× enhanced activity at 2 to 4 M NaCl and functioning at 40 MPa [198]). This enzyme also displayed increased activity when dissolved in 70% ethanol or n-propanol and extraordinarily high enantioselectivity in hydrolysis and transesterification of compounds important in the pharmaceutical, cosmetic, and food industries [198,245]. Thus, DHABs seem to be a suitable habitat for mining esterases which are potentially useful for industrial biotransformation, considering the great size of the lipolytic enzyme market, which is valued at the billion-dollar mark in the world’s market [246].

A study conducted on bacterial strains isolated from haloclines of Urania, Bannock, Discovery, and L’Atalante showed that *Bacillus horneckiae* gave highly stereoselective reduction for racemic propyl ester of anti-2- oxotricyclo[2.2.1.0]heptan-7-carboxylic acid (R,S)-1, a key intermediate of the synthesis of D-cloprostenol (chemical analog of prostaglandin [66]). Another isolate of *Halomonas aquamarina* was found to enantioselectively hydrolyze this molecule, indicating the potential of DHAB extremophile microbiome and marine-derived esterases and ketoreductases in stereoselective biocatalysis [66]. The same authors also isolated *Bacillus lehensis* from Discovery DHABs which harness an alkali-tolerant cyclodextrin glycosyltransferase and are able to produce non-toxic products for the pharmaceutical, cosmetic, and food industries. 

Overall, DHABs seem to be a suitable habitat for mining novel biocatalyst enzymes which are potentially useful for industrial biotransformations, encouraging further scientific challenges and research for fully realising the potential of DHAB extremozymes.

### 3.3. DHAB-Derived Prokaryotes: Promising Candidates for Enhanced Bioremediation of Oil Hydrocarbons

Petroleum hydrocarbons are among the most widespread pollutants on our planet and are becoming a severe problem because of their causing harmful damage to the environment and human health [247,248]. Oil pollution can occur in the environment following either catastrophic accidents (shipping disasters or pipeline failures) or natural oil seepages and biota [249]. Such contaminants can exert carcinogenic, neurotoxic, and mutagenic effects when organisms are exposed to them, significantly impacting the environment [250,251,252]. For these reasons, many innovative technologies have been developed for the clean-up of oil-polluted areas [247]. One of the most reliable of these is certainly bioremediation, which exploits the metabolic capabilities of microorganisms to break down recalcitrant hydrocarbons into harmless by-products, thus minimising the impact on the environment [253]. It is a more environmentally friendly alternative when compared with classical remediation techniques, which allow the reduction from the environment of a vast array of pollutants [254]. 

Most petroleum hydrocarbons encountered in the environment can be degraded or metabolized by indigenous bacteria which have developed specific pathways for sustaining their energetic and carbon requirements for living and blooming in the presence of these contaminants [255,256]. Indeed, many studies have focused their attention on hydrocarbon-degrading bacteria in oil-rich environments, including oil spill areas and oil reservoirs [257], and have demonstrated that their abundance is closely related to the respective types of petroleum hydrocarbons and surrounding environmental factors [258,259,260,261]. Despite this, many other normal and extreme microorganisms have been isolated and employed as biodegraders for dealing with petroleum hydrocarbons, representing a promising biotechnological alternative for achieving oil-hydrocarbon degradation [9,262]. Because of the particular physiological characteristics of microorganisms isolated from extreme environments, including DHABs, prokaryotes can be employed for enhanced bioremediation of oil hydrocarbons, especially in hypersaline environments [263,264]. For instance, members of the genera *Alcanivorax* and *Marinobacter,* which have been isolated respectively from Erba halocline and Shaban Deep [70,84], are essential marine hydrocarbonoclastic bacteria present in the active phase of oil spills, playing a significant role in the natural remediation of oil-polluted marine environments all over the world [265,266,267]. Their number increases very quickly after oil spills, although it declines only a few weeks later (see [268] as well as references therein). The outstanding bioremediation capacity of *A. dieselolei* has also been supported by sequencing the genome of the strain KS-293 isolated from surface seawater [269]. Its genome consistently contains multiple genes and enzymes involved in pathways associated with hydrocarbon degradation (linear and branched alkanes) and shows high similarity with *A. dieselolei* strain B5 [270,271,272]. This strain has been observed to preserve cell integrity under pressures of up to 10 MPa cultured with n-dodecane as a sole carbon source, downregulating 95% of its genes [273,274]. Additionally, Sass et al., 2008 demonstrated that the strain DS-1, closely related to *Bacillus aquimaris*, isolated from the Discovery DHAB, could grow with n-alkanes (n-dodecane and n-hexadecane) in the presence of 12–20% NaCl [275]. Furthermore, *Salinisphaera shabanensis* and *Marinobacter salsuginis* have been isolated from the Shaban Deep, displaying a high capacity to assimilate aliphatic hydrocarbons [70,276]. *S. shabanensis* can be cultured at a wide range of salinity and temperatures (0.2–4.8 M NaCl and 5–42 °C), on a vast array of substrates including n-alkanes (dodecane). *M. salsuginis* is a heterotrophic, facultative anaerobic bacterium capable of fermentation and nitrate reduction [70].

Moreover, other bacteria belonging to the genus *Marinobacteria* and isolated from seawater and Nereus halocline have shown to be efficient for bioremediation purposes for degrading hydrocarbons, including polycyclic aromatic hydrocarbons (PAHs), as revealed by the complete genome sequence of *Marinobacter flavimaris* SW-145 [277,278,279,280]. Other strains belonging to the genera *Vibrio*, *Pseudomonas*, *Arthrobacter* *Pseudoalteromonas*, *Idiomarina*, *Halomonas,* and *Thalassospira* identified in different DHABs have been collected and cultured from marine sediments and are able to grow on PAHs including naphthalene, dibenzothiophene, pyrene, and phenanthrene [21,39,267,281,282]. 

Even though many oil hydrocarbons can be easily degraded in low salinity marine habitats [267], very little is known about their fate in moderate and in hypersaline environments where microbial activity is enormously inhibited [263,283]. To this purpose, some archaea belonging to the class of *Halobacteria* identified in many DHABs hold great promise and have considerable potential to bioremediate hydrocarbons in high salty environments such as nearshore oil production sites, salt marshes, sabkhas, and other coastal flats, including industrial wastewaters [264,284].

Haloarchaea of the class Halobacteria identified in many DHABs located both in the Mediterranean and the Red Sea [17,44,56,189] can produce PAH-degrading enzymes, which may be exploited to remove aromatic hydrocarbons from the polluted environments safely [285]. In the hypersaline coastal areas of Kuwait, hydrocarbonoclastic haloarchaea, together with *M. flavimaris,* a diazotrophic strain able to grow under 1 M–3.5 M NaCl conditions, have effectively contributed to oil bioremediation [286].

Although more archaeal strains have been isolated, our information on the physiological, biochemical, and genomic basis of hydrocarbon degradation by members of the *Halobacteria* is still extremely scant [263,287]. Such information is crucial for designing novel and more efficient technologies employing haloarchaea for the remediation of contaminated high salinity environments. 

## 4. Conclusions and Future Directions 

Polyextremophilic bacteria and archaea are an extraordinary reservoir of novel enzymes and bioactive molecules which can provide important benefits for different biotechnological applications, ranging from medicine to environmental fields. So far, studies on DHABs are limited, and we urgently need to expand data on microbial diversity and ecology of these extreme ecosystems. The uniqueness of these habitats is able to select for highly specialized organisms which show extreme adaptions at morphological, physiological, biochemical, and genetic levels, hinting at a bright future for “blue” biotechnology. In this extensive literature review we have observed that polyextremophiles maintain several high metabolic similarities to other non-extreme prokaryotes. This important issue, which remains to be further investigated, could open new research perspectives on the production of the biomolecules’ portfolio of marine microorganisms. Such explorations are expected to provide huge rewards not only in terms of the impact on existing industries for the discovery of new products with beneficial or useful properties, but also in the “blue” economy. This scenario is also perfectly framed within the Sustainable Development Goals of the United Nations, which aims to identify actual solutions for disease outbreaks, climate change, and environmental degradation in order to have safer, cleaner, and more efficient industrial manufacturing processes in order to improve human health and wellbeing from a sustainable development perspective.

Nowadays, “blue” biotechnologies are taking advantage of increasing numbers of “-omics” tools and high-throughput screenings for unveiling the chemical diversity of the extreme environments present in the oceans. These tools are facilitating the identification of prokaryotic metabolic adaptations, which can lead to the production of novel molecules and thus can be exploited for the development of new biotechnologies. To date, novel uncultured species identified in DHABs of the genera *Streptomyces*, *Pseuodalteromonas*, and *Bacillus* seem to hold great potential in producing new bioactive molecules. Among the culturable species identified in DHABs, *Chromohalobacter israelensis, Zunongwangia profunda, Marinobacter flavimaris, Alcanivorax dieselolei, Halomonas meridiana, Alteromonas macleodii,* and *Bacillus halodurans* are promising species for biotechnological applications. Further innovative technologies and studies applied to DHABs will be essential to carry out in-depth investigations and to disentangle microbial assemblages, functions, and metabolites of biotechnological interest from these peculiar systems. Thus, the actual development of DHAB-derived biotechnologies will depend on technological and methodological advancements and the ability of scientists to promote research projects for the study of these ecosystems. 

## Figures and Tables

**Table 1:** Minimum and maximum values of the main physicochemical variables observed among DHABs.

Environmental Parameters	Ranges	DHABs	Location	References
Temperature	Min: 14 °C Max: 68 °C	La Medee Atlantis II	Mediterranean Sea Red Sea	[31] [32]
Depth	Min 630 m Max: 3580 m	GC233 Discovery	Gulf of Mexico Mediterranean Sea	[33] [17]
Na^+^	Min: 1751 mM Max 5300 mM	GC233 Tyro	Gulf of Mexico Mediterranean Sea	[24] [34]
Cl^-^	Min: 2092 mM Max: 10,154.3 mM	GC233 Discovery	Gulf of Mexico Mediterranean Sea	[24] [17]
Mg^2+^	Min: 8.7 mM Max: 5143 mM	GB425 Discovery	Gulf of Mexico Mediterranean Sea	[24] [17]
K^+^	Min: 17.2 mM Max: 471 mM	Orca La Medee	Gulf of Mexico Mediterranean Sea	[35] [31]
Ca^2+^	Min: 1 mM Max: 150 mM	Discovery, Kyros Atlantis II	Mediterranean Sea Red Sea	[17] [36]
SO_4_^2−^	Min: <1 mM Max: 333.1 mM	GB425; GC233 L’Atalante	Gulf of Mexico Mediterranean Sea	[24] [17]
Sulfide	Min: 0.002 mM Max: 16 mM	GC233 Urania	Gulf of Mexico Mediterranean Sea	[24] [16]

**Table 2:** Bioactive molecules for pharmaceutical use produced by prokaryotes directly isolated from DHABs and promising bioactive molecules produced by prokaryotic taxa which have been identified in DHABs and isolated from other marine systems.

Marine Prokaryotes	Product	Bioactivity	Environmental Sources	Ref.
*Alteromonas macleodii*	Dithiolopyrrolone	Antibiotic and antitumor	Erba and Nereus DHABs	[84,104]
*Alteromonas sp*. B-10-31	Marinostatins B-1, C1, and C2	Serine protease inhibitor	Coastal seawater	[105]
*Bacillus sp.*	Macrolactins A–F	Cytotoxic, antimicrobial, antiviral	Deep sea	[106,107,108,109]
*Bacillus halodurans*	Enfuvirtide	Antiviral	Nereus DHAB	[84,110]
*Bacillus* MK-PNG-276A	Loloatins A–D	Antimicrobial	Great barrier reef	[111]
*Bacillus sp.*	Bogorol A	Antimicrobial	Seawater	[112]
*Bacillus sp.* CND-914	Halobacillin	Antitumor	Deep-sea sediments	[113]
*Bacillus sp.* MIX-62	Mixirins A–C	Antitumor		[114]
*Bacteroidetes rapidithrix* HC35	Ariakemicins A and B	Antimicrobial, cytotoxic	Sea mud	[115]
*Erythrobacter sp*.	Erythrazoles A and B Erythrolic acids A–E	Cytotoxic	Mangrove sediments	[116,117]
*Halobacteroides lacunaris* TB21	R-LPS	Immunomodulator	Thetis DHAB	[118]
*Halomonas* LOB-5	Loihichelins A–F	n.a.	Deep sea hydrothermal vents	[119]
*Halomonas* meridiana	n.a.	Antitumor	Nereus DHAB	[84]
*Halomonas sp*. GWS-BW-H8hM	3-(4′-Hydroxyphenyl)-4-phenylpyrrole-2,5-dicarboxylic acid (HPPD-1 and HPPD-2)	Cytotoxic	Seawater	[120]
*Halomonas* sp. GWS-BW-H8hM	2-Amino-6-hydroxyphenoxazin-3-one2-Amino-8-benzoyl-phenoxazin-3-one2-Amino-8-(4-hydroxybenzoyl)-6-hydroxyphenoxazin-3-one	Antimicrobial, cytotoxic	Seawater	[121]
*Pseudoalteromonas carrageenovora* IAM 12662	LPS	Antiviral	Erba DHAB	[84,122]
*Pseudoalteromonas haloplanktis* TAC125	Peptides	Antioxidant	Antarctic coastal sea water	[123]
*Pseudoalteromonas mariniglutinosa*	n.a.	Antitumor	Erba and Nereus DHABs	[84]
*Pseudoalteromonas rava* SANK 73390	Thiomarinols A–H and J	Antimicrobial	Seawater	[124,125]
*Streptomyces aureoverticillatus* (NPS001583)	Aureoverticillactam	Antitumor	Marine sediments	[126]
*Streptomyces* C42	Champacyclin	Antimicrobial	Deep sea	[127]
*Streptomyces* CNH-990	Marmycins A and B	Cytotoxic	Seawater	[128,129]
*Streptomyces drozdowiczii* SCSIO 10141	Marfomycins A, B, and E	Anti-infective	Deep sea	[130]
*Streptomyces drozdowiczii* NTK 97	Frigocyclinone	Antimicrobial	Antarctica	[131]
*Streptomyces Merv* 8102	Essramycin	Antimicrobial	Marine animals, plants, and sediments	[132]
*Streptomyces niveus* SCSIO 3406	Marfuraquinocins	Cytotoxic antimicrobial	Deep sea	[133]
*Streptomyces scopuliridis* SCSIO ZJ46	Desotamide B	Antimicrobial	Deep-sea sediments	[134]
*Streptomyces sioyaensis* SA-1758	Altemicidin	Cytotoxic, antimicrobial	Sea mud	[135]
*Streptomyces sp*. 12A35	Lobophorins H and I	Antimicrobial	Deep sea	[136]
*Streptomyces sp*. ART5	Articoside	Cytotoxic,	Arctic deep sea	[137]
*Streptomyces sp*. CNB-982	Cyclomarins A–C	anti-inflammatory	Marine sediments	[138,139]
*Streptomyces sp*. CNQ-418	Marinopyrroles A–F	Antimicrobial, cytotoxic, anti-apoptotic	Deep-sea sediments	[140,141]
*Streptomyces sp*. CNQ-85	Daryamides A–C (2E,4E)-7-Methylocta-2,4-dienoic acid amide 26	Antitumor, antifungal	Seawater	[142]
*Streptomyces sp*. CNR-698	Ammosamides A–D	Cytotoxic	Deep sea	[143,144,145]
*Streptomyces sp*. M045	Chinikomycins A and B	Antitumor	Seawater	[146]
*Streptomyces sp*. MDF-04-17-069	Tartrolon D	Cytotoxic	Marine sediments	[147]
*Streptomyces sp*. Mei37	Mansouramycins A–D	Antimicrobial, cytotoxic	Marine sediments	[148]
*Streptomyces sp*. NTK 935	Benzoxacystol	Antiproliferative	Deep sea	[149]
*Streptomyces sp*. SCSIO 03032	Spiroindimicins A–D	Antitumor	Deep sea	[150]
*Streptomyces sp*. SCSIO 11594	Dehydroxyaquayamycin, Marangucycline B	Antibacterial, antitumor	Deep sea	[151]
*Streptomyces xiamenensis* M1-94P	Xiamenmycin C and D	Anti-fibrotic	Deep-sea sediments	[152]
*Streptomycete sp*.	Piperazimycins A–C	Antitumor	Marine sediments	[153]
*Zunongwangia profunda* SM-A87	EPS	Antioxidant	Nereus DHAB	[154]

**Table 3:** DHAB microbiome as a source of polyextremozymes. The bacterial and archaeal species marked with an asterisk have been isolated from DHABs, whereas the other genera are potentially producers of extremozymes because these have identified from DHABs (but not cultured thus, being isolated from marine and/or other extreme environments).

Enzyme	Biological Source	Specific Adaptations	Function and/or Applications	Ref.
Aldehyde dehydrogenase (EC 1.2.1.3–7)	*Bacillus halodurans* from Nereus interface; Atlantis II Red Sea brine pool; Cytophaga sp. KUC-1 from Antarctic seawater and *Halobacterium salinarum*	Slight halophile; thermo- and psychrophilic	Biotransformation of a large number of drugs and other xenobiotics generates aldehydes as intermediates or as products resulting from oxidative deaminations	[172,173,174,175]
Protease (EC 3.4.21–25)	Salinivibrio costicola* and Pseudoalteromonas ruthenica* from Erba DHAB. *Bacillus circulans* BM15 and *PseudoAlteromonas sp*. 129-1. *Bacillus sp.* NPST-AK1, *Halobacterium halobium* (ATCC 43214), *Bacillus licheniformis*, *Bacillus halophilus*, *Pseudoalteromonas strain* EB27, *Halomonas meridiana* DSM 5425, *Bacillus sp.* (Ve2-20-91 (HM047794)), and *Bacillus caseinilyticus*	Haloalkaliphilic and thermotolerant alkaline	Protein hydrolysis finds a broad variety of potential applications in diverse biotechnological processes such as in the feed, food, pharmacology (anticancer and antihemolytic activity) and cosmetic (keratin-based preparation) industries, and cleaning processes (e.g., detergent additive)	[176,177,178,179,180,181,182,183,184,185]
Cellulase (EC 3.2.1.4)	Cytophaga hutchinsonii, Halorhabdus tiamatea from Shaban DHAB, *Bacillus sp.* SR22 from seawater, *Bacillus sp.*, *Vibrio sp*., *Rhodococcus sp*., *Clostridium* and *Streptomyces* from mangrove Halorhabdus utahensis from Great Salt Lake	Halo-alkali tolerant and thermotolerant	Breakdown of cellulose-producing polysaccharides; potential application in the food, animal feed, beer and wine, textile and laundry, and pulp and paper industries, agriculture, biofuel, pharmaceutical industries, and waste management	[186,187,188,189,190,191]
Chitinase (EC 3.2.1.14), chitin deacetylase (EC 3.5.1.41)	*Bacillus thuringiensis* HBK-51 from soil. *PseudoAlteromonas sp*. DC14, *Vibrio cholerae, Vibrio parahaemolyticus*, and *Arthrobacter sp*. AW19M34-1 from seawater	Halo-alkali tolerant and thermotolerant	Hydrolysis of chitin and hence N-acetyl chitobiose production which in turn can be useful in fermentation research and biomedicine. There have also been applications in the cosmetic and pharmaceutic fields	[192,193,194]
Esterase (EC 3.1.1.1)	Zunongwangia profunda* from Atlantis II and Nereus interface and brine pools. *Alcanivorax dieselolei* B5(T) from Erba interface. *Bacillus cereus* AGP-03 from hot spring. *Archaeoglobus fulgidus*	Thermo-halotolerant and metal resistant; cold-active and organic solvent-tolerant	Leather manufacturing, flavor development in the dairy industry, oil biodegradation, and the synthesis of pharmaceuticals and chemicals	[195,196,197,198,199,200]
Ferredoxin oxidoreductase (EC 1.2.7.1)	*Halorhabdus tiamatea* SARL4BT* from Shaban DHAB. *Desulfovibrio sp*. from Atlantis II DHAB. Methanosarcina barkeri	Low-oxygen tolerant	Oxidation/reduction processes which are applied in the asymmetric oxyfunctionalization of steroids and other pharmaceuticals, synthesis and modification of polymers, oxidative degradation of pollutants, oxyfunctionalization of hydrocarbons, and the construction of biosensors for diverse clinical applications	[189,201,202]
Lipase (EC 3.1.1.3)	Idiomarina sp. W33, Halo*Bacillus sp.*, and *Archaeoglobus fulgidus*. *Marinobacter alkaliphilus* ABN-IAUF-1. *Bacillus sp.*, *Arthrobacter sp*., *Pseudomonas sp*., and *Psychrobacter sp*. from Antarctic marine sediments. Oceano *Bacillus sp.* PUMB02 from seawater	Halo- alkalitolerant and hyperthermophilic	Hydrolysis of acylglycerols to release fatty acids and lower acylglycerols or glycerol. Lipase enzymes are exploited in the food, beverage, detergent, biofuel production, animal feed, textiles, leather, paper processing, and cosmetic industries	[203,204,205,206,207,208]
Mercuric reductase (EC 1.16.1.1)	Atlantis II deep-sea brine. Chromohalobacter israelensis* from Erba and Atlantis II DHABs. Bacillus firmus* from Discovery DHAB	Extreme halophilic and thermophilic	This enzyme can convert toxic mercury ions into relatively inert elemental mercury. It is very useful in waste-water treatments	[209,210]
Nitrilase (EC 3.5.5.1)	Red Sea Atlantis II brine	Thermostable and heavy metal tolerant	Nitrilase can hydrolyze a single cyano group in dinitriles or polynitriles, yelding cyanocarboxilic acids, which are used in different kinds of industries, including the food and pharmacology industries; also used for bioremediative purposes	[211]
Pullulanase (EC 3.2.1.41)	*Bacillus sp.* and *Streptomyces sp*.	Alkaliphilic	Utilized to hydrolyze the α-1,6 glucosidic linkages in starch, enabling a complete and efficient conversion of the branched polysaccharides into small fermentable sugars during the saccharification process	[212]
Xylanase (EC 3.2.1.8) and β-Xylosidase (EC 3.2.1.37)	*Staphylococcus sp., Arthrobacter sp*., *Streptomyces* sp., and *Vibrio sp*. XY-214 from seawater. Oceanospirillum linum CL8 and *Halorhabdus utahensis* from Great Salt Lake. *Halorhabdus tiamatea* SARL4BT* from Shaban DHAB. Pseudoalteromonas mariniglutinosa* from Erba and Nereus DHAB. Marinimicrobium haloxylanilyticum* from Kebrit DHAB. Zunongwangia profunda* from Nereus and Atlantis II DHABs. Halomonas meridiana* from Bannock, Erba, and Nereus DHABs. Bacillus halodurans* from Nereus interface	Alkali-halotolerant and psychrophilic	Commercial exploitation in the areas of the food, feed, and paper and pulp industries; also used to increase sugar recovery from agricultural residues for biofuel production	[189,213,214,215,216,217,218]
α-agarase (EC 3.2.1.158) and β-agarase (EC 3.2.1.81)	Alteromonas macleodii* from Erba, Discovery, and Nereus DHABs. *Alteromonas sp*. GNUM-1, *Alteromonas agarlyticus*, *Alteromonas sp*. strain C-1, *Vibrio sp*. PO-303, *Altermonas sp*. SY37-12, and Cytophaga flevensis from seawater and marine sediments	Moderate halophile	Degradation of agar-degrading bacteria used as oriental food; wide applications in the food industry, cosmetics, and medical fields, and as a tool enzyme for biological, physiological, and cytological studies	[219,220,221]
α-amylase (EC 3.2.1.1)	PontiBacillus chungwhensis* from Discovery DHAB. Halomonas meridiana* from Nereus, Erba, and Bannock DHABs. Zunongwangia profunda* from Atlantis II and Nereus DHABs. *Cytophaga sp*. *HaloBacillus sp.*, *Bacillus sp.* GM8901, *Bacillus sp.* TSCVKK, and *Methanococcus jannaschii. Halobacterium sp*. from hypersaline environment. *Alteromonas haloplanctis* from Antarctic seawater	Moderate halophile and alkali- tolerant; hyperthermophilic	α-amylase has implications in the food, pharmaceutical, and chemical industries; multifunctional amylase exhibits transglycosylation and hydrolysis activities to produce isomaltooligosaccharides, maltooligosaccharides and glucose	[222,223,224,225,226,227,228,229,230,231,232]
β-glucosidases (EC 3.2.1.21)	*Halorhabdus tiamatea* SARL4BT* from Shaban DHAB. *Alteromonas sp*. L82 from the Mariana Trench. Cytophaga hutchinsonii	Low-oxygen tolerant, cold-adapted, and salt-tolerant	β-glucosidases convert cellobiose and short cellodextrins into glucose. β-glucosidases are widely used in the production of biofuels and ethanol from cellulosic agricultural wastes, in the production of wine, and in the flavor industry. They can cleave phenolic and phytoestrogen glucosides from fruits and vegetables for extracting medicinally important compounds and enhancing the quality of beverages	[189,191,233,234]
κ-Carragenases (EC 3.2.1.83)	Pseudoalteromonas carrageenovora* from Erba sediments. *Bacillus sp. Alteronomonas sp*., Cytophaga sp., and *PseudoAlteromonas sp. Pseudomonas sp*., *Vibrio sp*. NJ-2, and *Vibrio parahaemolyticus* from seawater	Alkali-halotolerant	Production of oligosaccharides with potential applications in the biomedical field, in bioethanol production, in the textile industry, and as a detergent additive	[235,236,237]
Cyclodextrin glycosyltransferase (EC 2.4.1.19)	Bacillus lehensis* from Discovery DHAB	Alkali-halotolerant	Cyclodextrins produced by this enzyme have broad, non-toxic applications in the pharmaceutical, cosmetic, and food industries	[66,238]

## Supplementary Materials

The following information is available online at https://www.mdpi.com/1660-3397/18/2/91/s1, Table S1: Prokaryotic strains isolated from DHABs, Table S2: Prokaryotic strains isolated from DHABs and their closest relatives obtained from phylogenetic analyses.
